# Supplementary material for: Antimicrobial Biomaterial on Sutures, Bandages and Face Masks with Potential for Infection Control
Source: Polymers (Basel). 2022 May 10;14(10):1932. doi: 10.3390/polym14101932 (PMC9143446; doi:10.3390/polym14101932)
Supplement: Supplementary file 1 [file polymers-14-01932-s001.zip › Presentation Supplementary material.pdf]

Antimicrobial Biomaterial on Sutures, Bandages and Face Masks with Potential for Infection Control

Zehra Edis [1, 2,*], Samir Haj Bloukh [2,3] , Hamed Abu Sara [2,3] and Nur Izyan Wan Azalee [4]

Supplementary material in the article

Already added as pdf into zip file

Supplement S1-Bandage TCA area 1-coiled fibers part

And

Supplement S2-Bandage TCA area 2-ordered fibers part

Please see the provided pdf files.

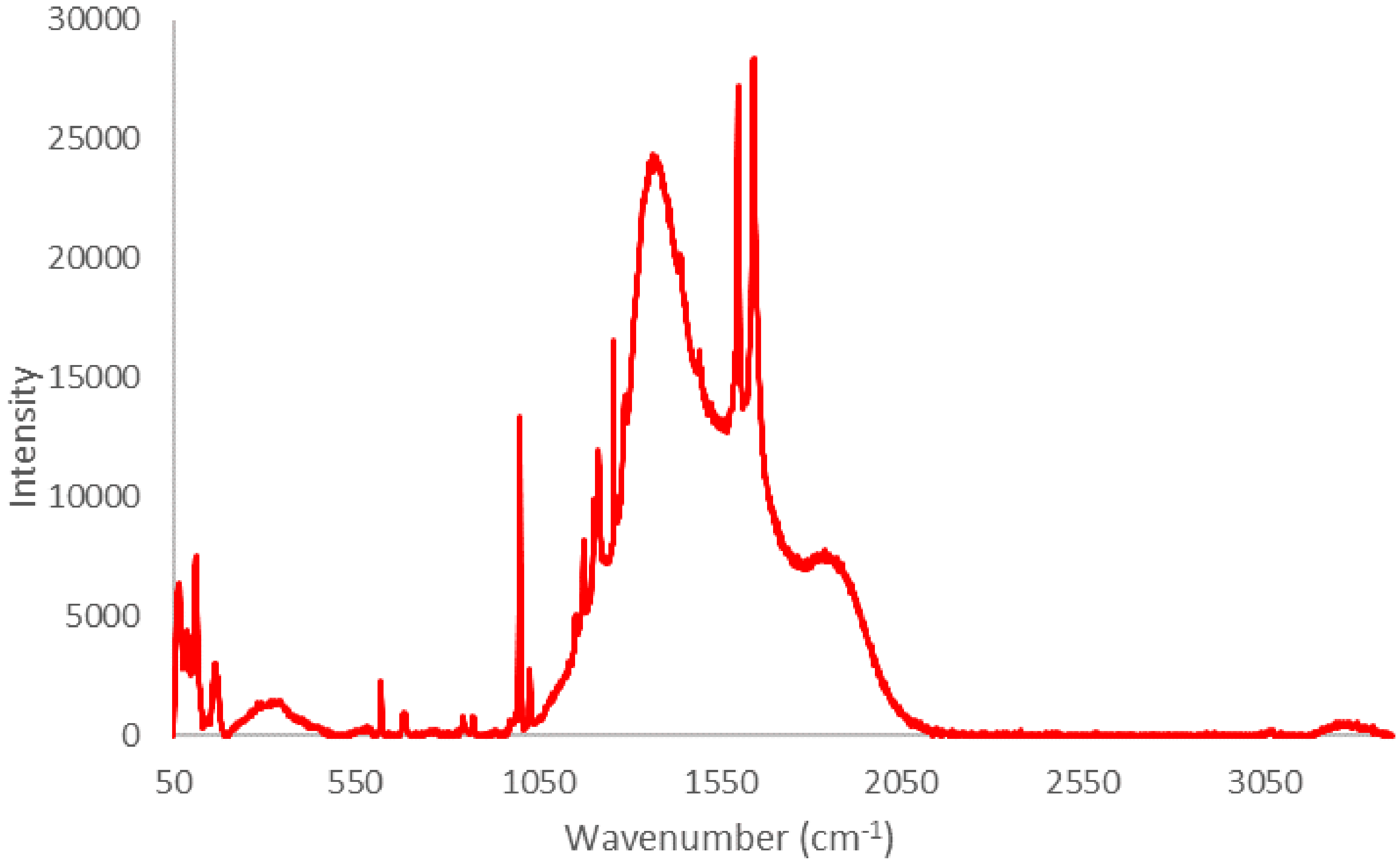

Supplement S3 Raman AV-PVP-TCA-I2

AJMAN UNIVERSITY

7/14/2020 1:56:13 PM

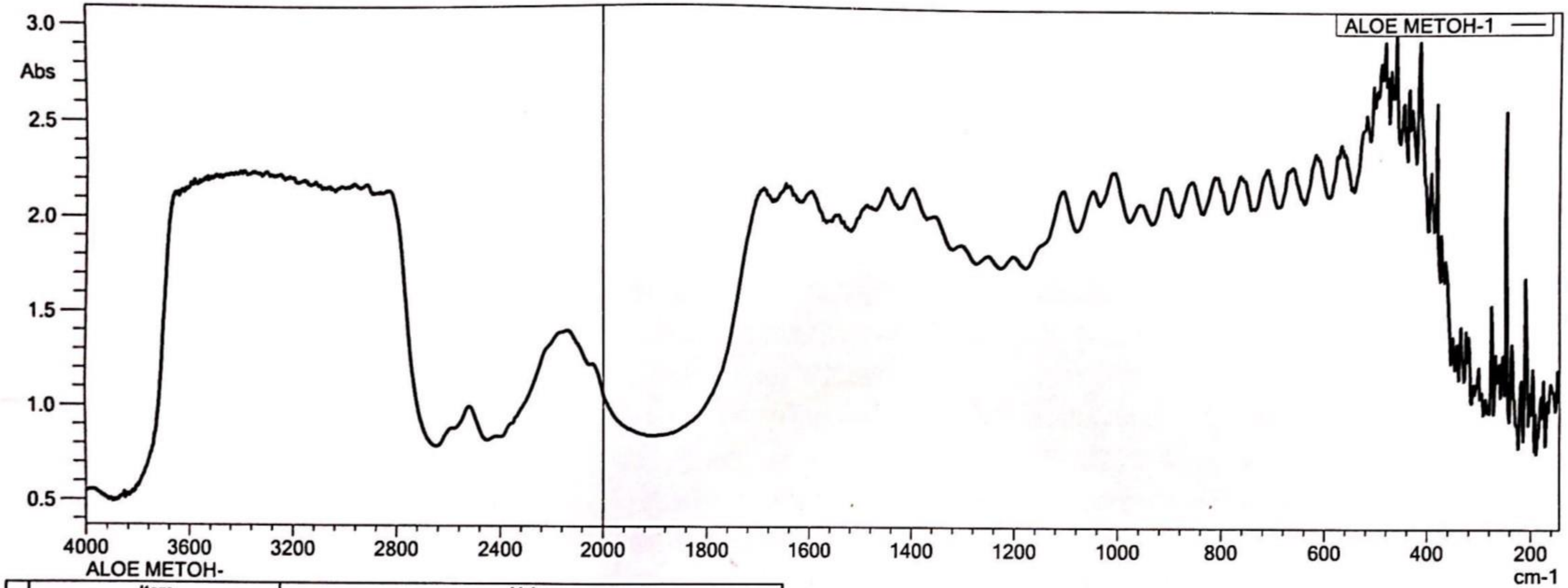

Item	Value
Acquired Date&Time	11/13/2019 1:01:38 PM
Filename	C:\Users\ADMIN\Desktop\Edis\ALOE METOH-1.ispd
Spectrum name	ALOE METOH-1
Sample name	Relyx ultimate pilot
Sample ID	
Comment	ALOE METOH-
No. of Scans	24
Resolution	4 [cm-1]

Supplement S4 AV FTIR

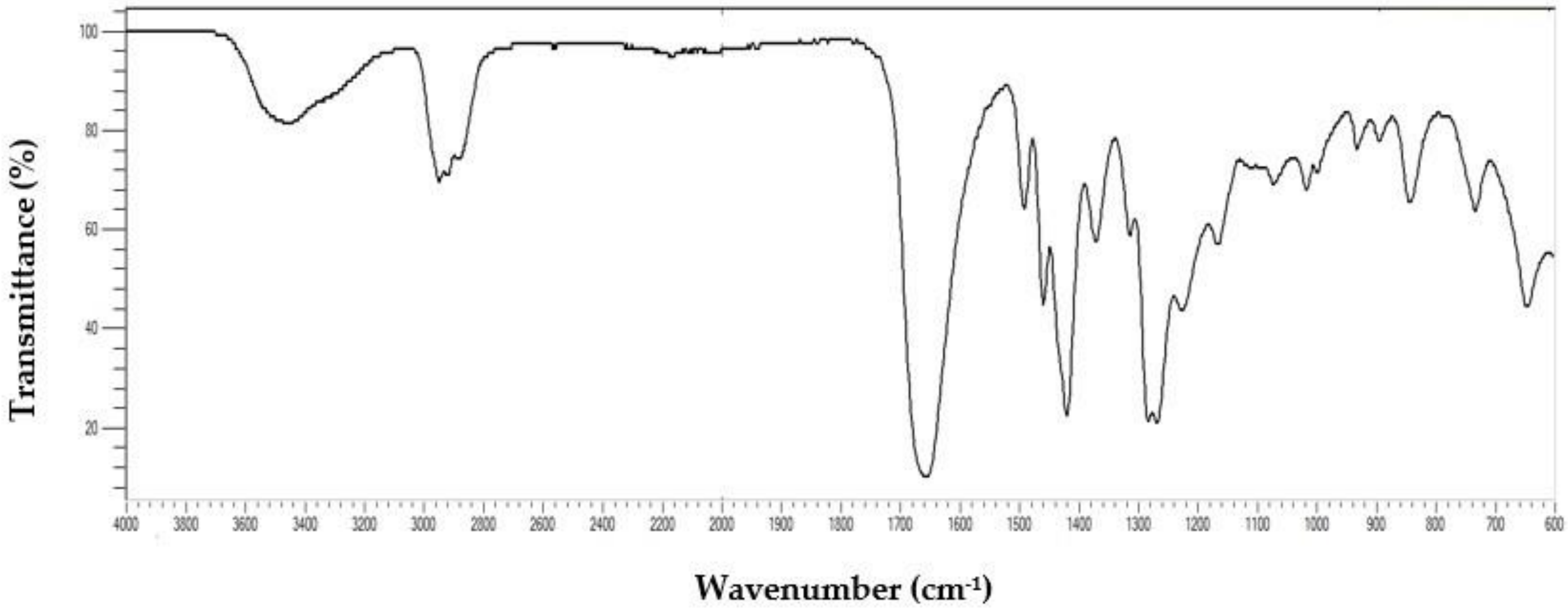

Supplement S5 PVPI FTIR
