# Supplementary figures and images for: Antimicrobial Biomaterial on Sutures, Bandages and Face Masks with Potential for Infection Control
Source: Polymers (Basel). 2022 May 10;14(10):1932. doi: 10.3390/polym14101932 (PMC9143446; doi:10.3390/polym14101932)

Electron Image 1

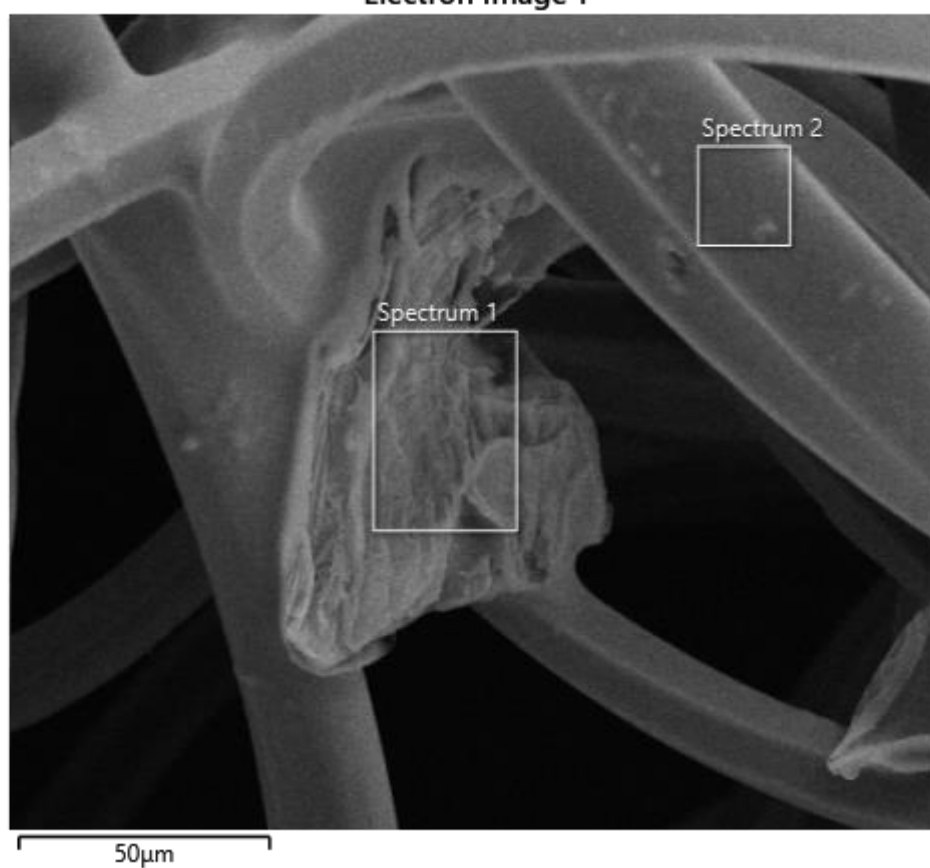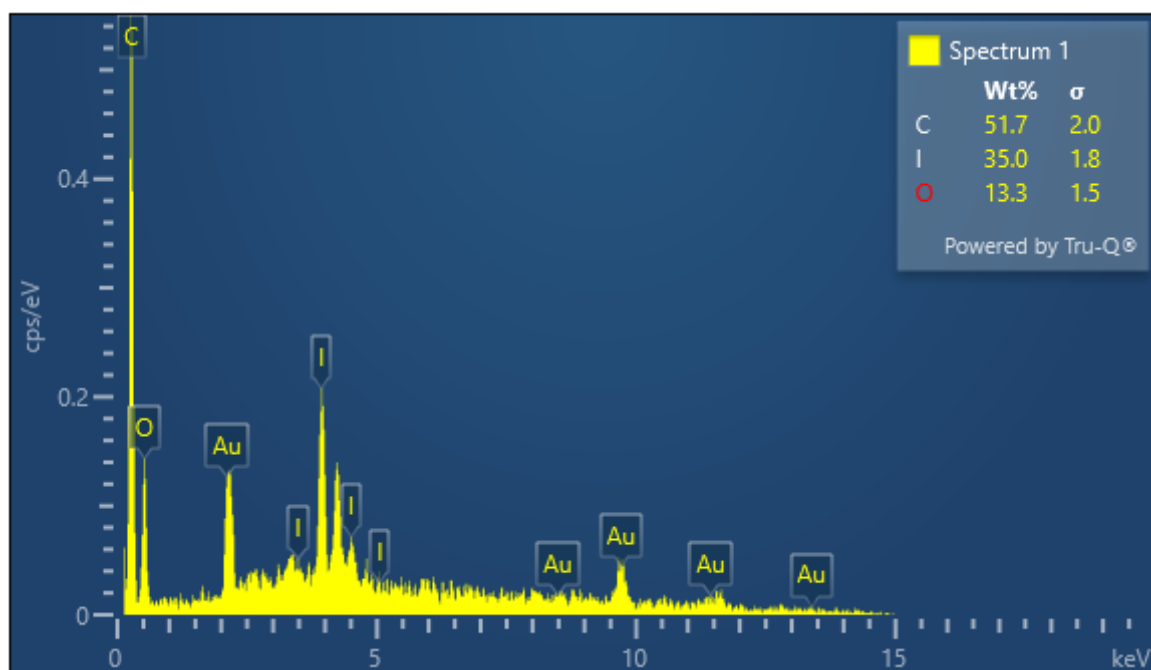

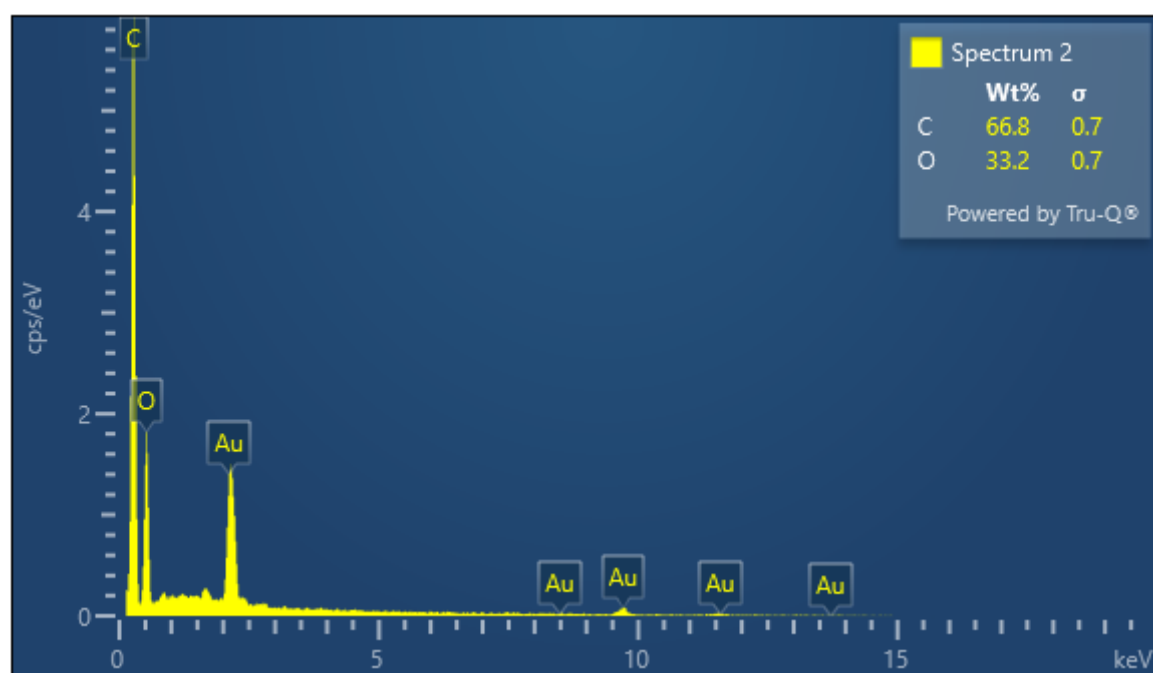

Supplement: Supplementary file 1 [file polymers-14-01932-s001.zip › Suplement Figure S1-Bandage TCA area 1-coiled fibers part.pdf]

Electron Image 7

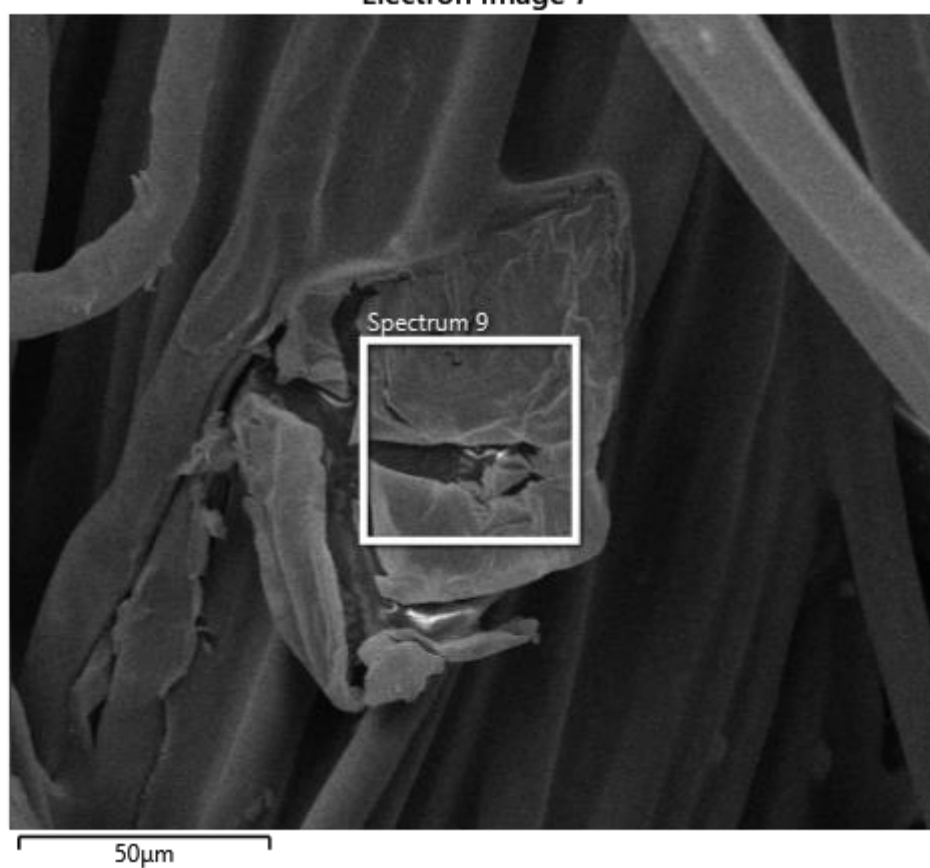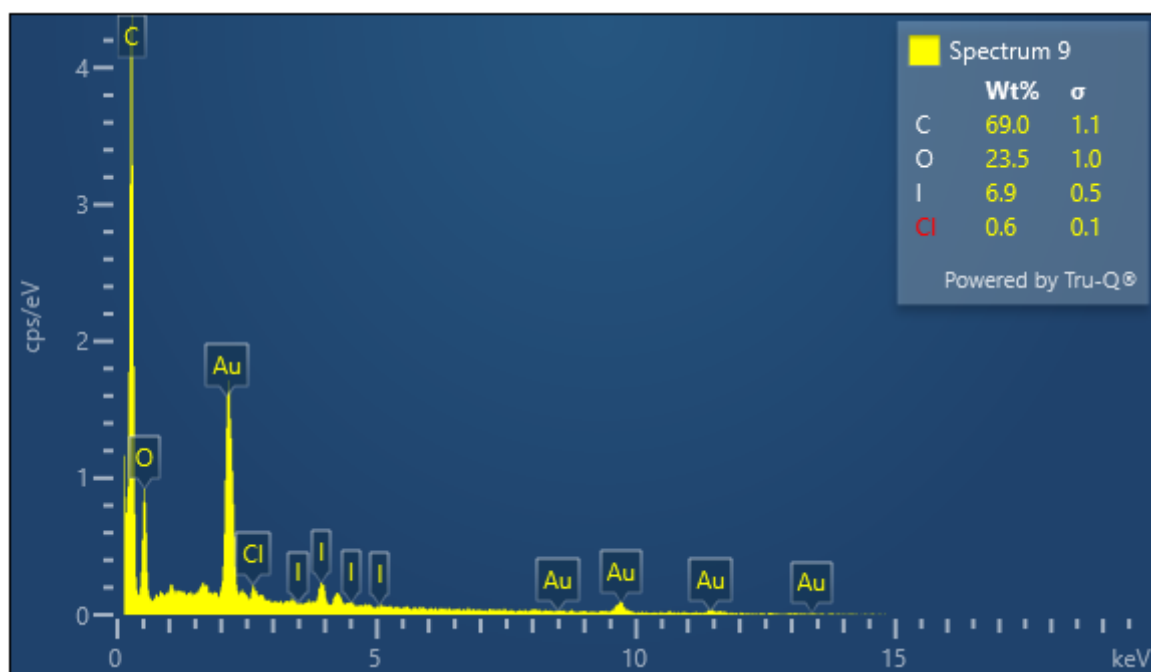

Supplement: Supplementary file 1 [file polymers-14-01932-s001.zip › Suplement Figure S2-Bandage TCA area 2-ordered fibers part.pdf]

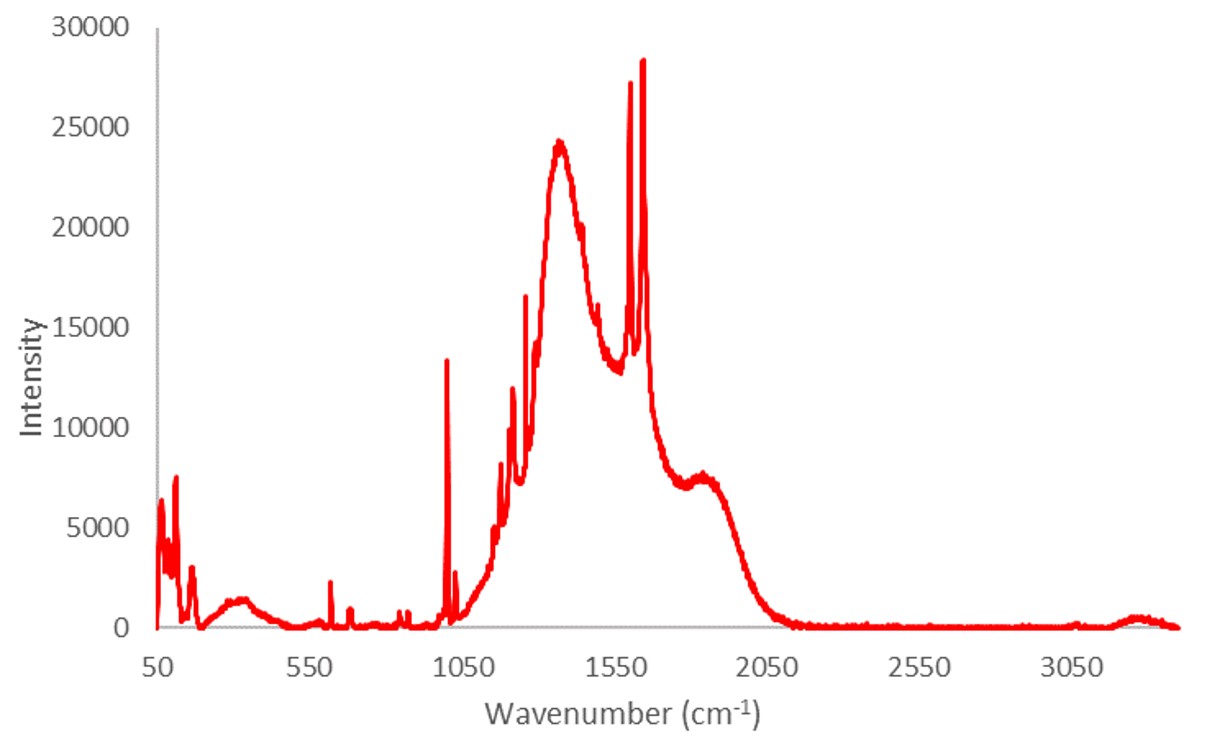

Supplement: Supplementary file 1 [file polymers-14-01932-s001.zip › Supplement Figure S3 Raman full new.jpg]

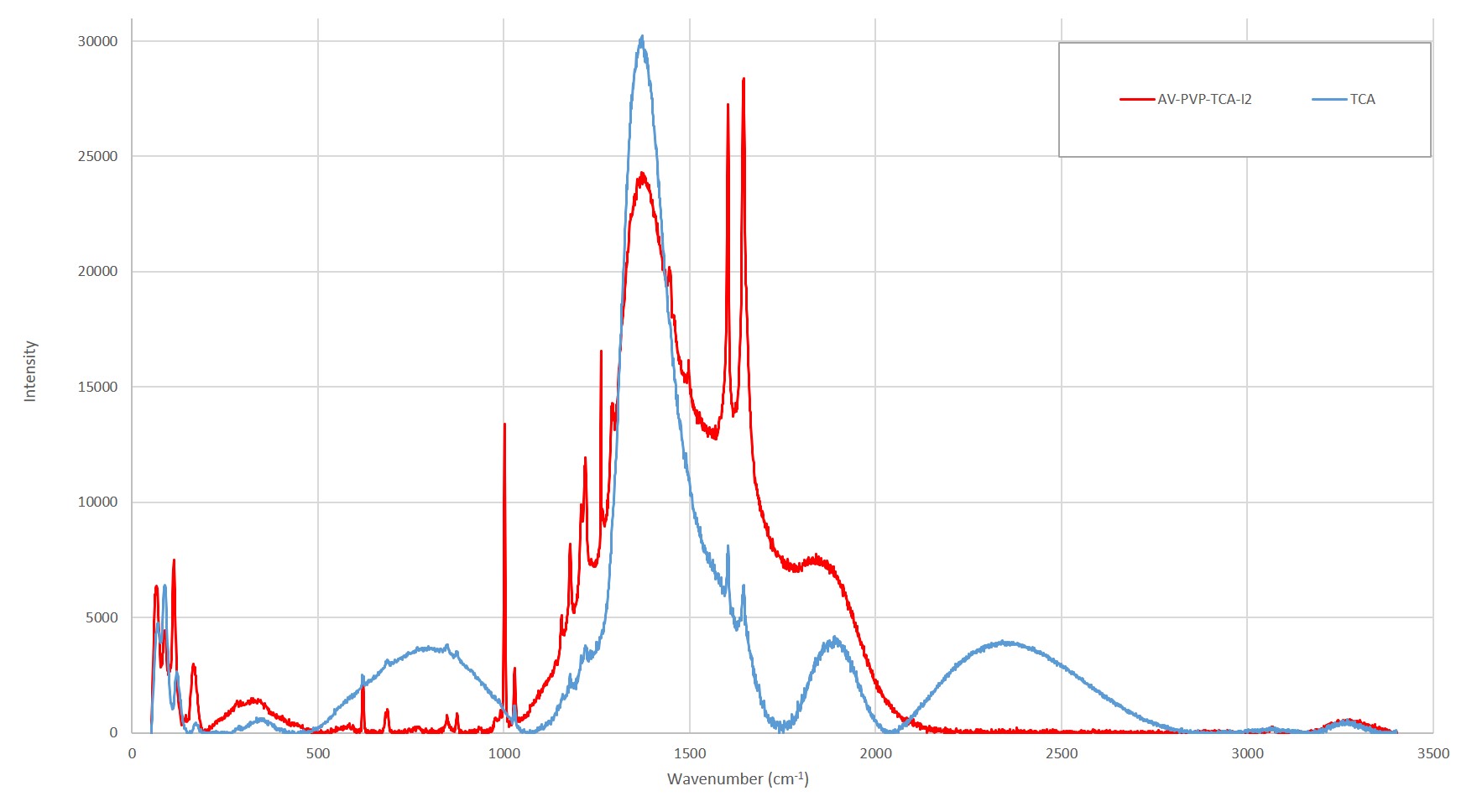

Supplement: Supplementary file 1 [file polymers-14-01932-s001.zip › Supplement Figure S3 Raman full w TCA.jpg]

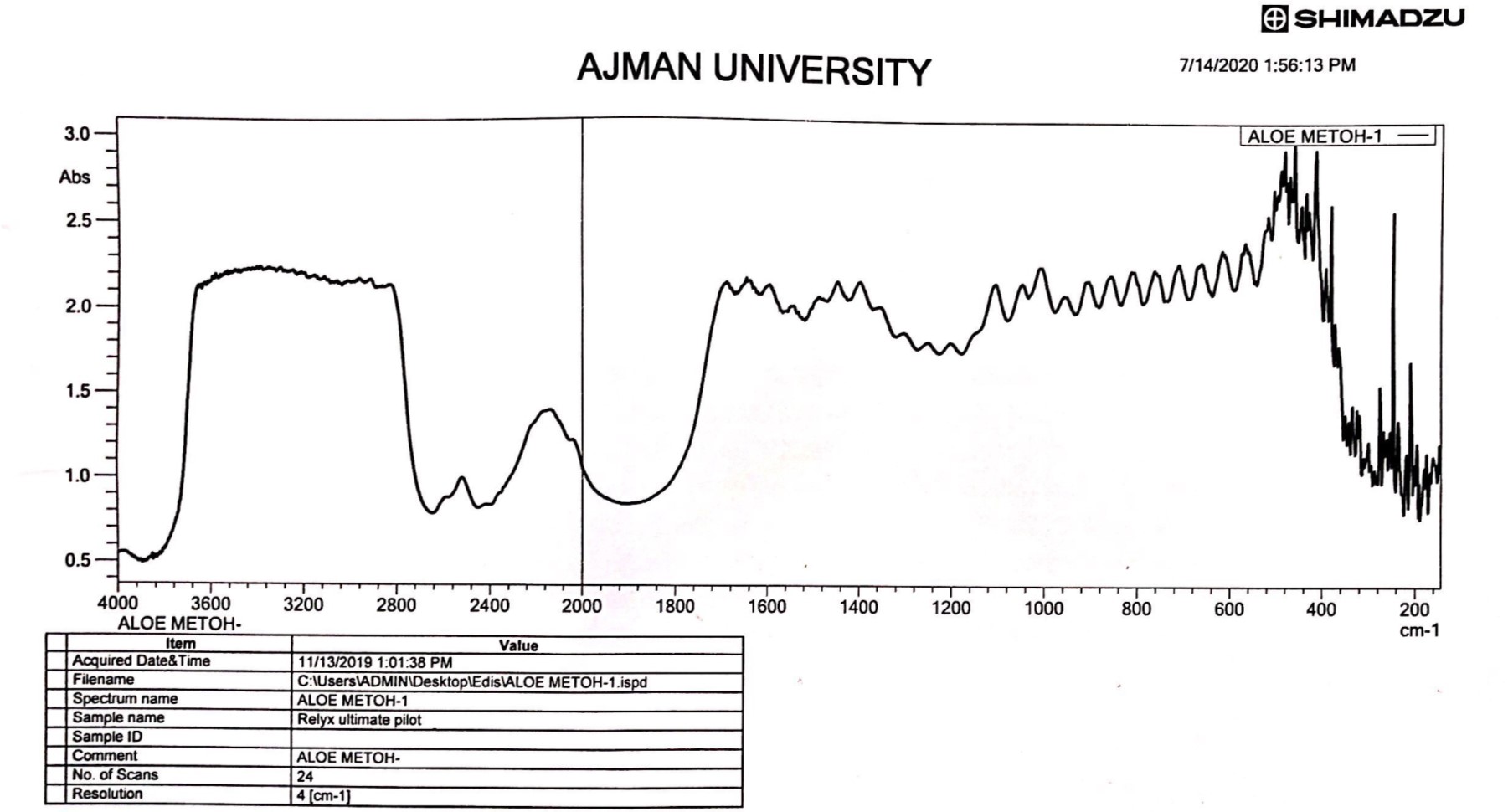

Supplement: Supplementary file 1 [file polymers-14-01932-s001.zip › Supplement Figure S4 AV FTIR.jpg]

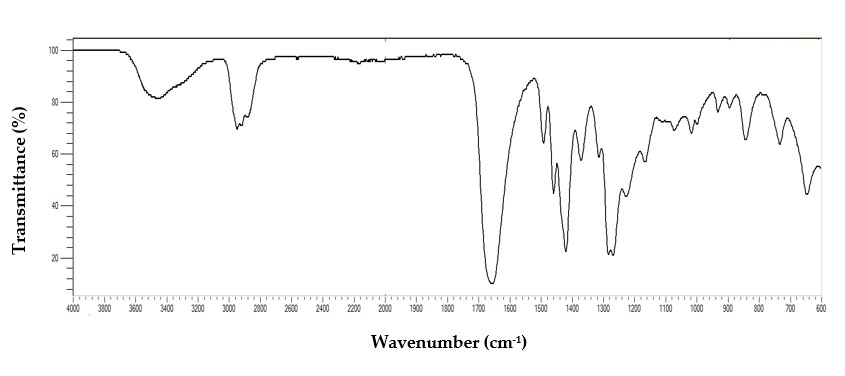

Supplement: Supplementary file 1 [file polymers-14-01932-s001.zip › Supplement Figure S5 PVPI FTIR.jpg]
